# Improving mariculture insurance premium rate calculation using an information diffusion model

**DOI:** 10.1371/journal.pone.0261323

**Published:** 2021-12-23

**Authors:** Qian Zhang

**Affiliations:** School of Business, Ningbo University, Ningbo, Zhejiang, China; Szechenyi Istvan University: Szechenyi Istvan Egyetem, HUNGARY

## Abstract

Mariculture is a well-known high-risk industry. However, mariculture insurance, which is an important risk management tool, is facing serious market failure. An important reason for this market failure lies in the unsound premium rate and pricing method. Due to a lack of long-term yield data, empirical rates are often adopted, but this adoption can lead to a high loss ratio. This paper provides an improved method for premium computation of mariculture insurance using an information diffusion model (IDM). An example of oyster insurance in China shows that, compared with the traditional pricing approach, the IDM can greatly improve the accuracy and stability of premium rate calculations, especially in cases of small samples.

## Introduction

With the growing global population and rapid economic growth, the consumption of global fishery products has been increasing [1]. It has increased from 9.0 kg/person in 1961 to 20.5 kg/person in 2016, with an annual growth rate twice that of the population growth rate, according to the Food and Agriculture Organization (FAO). However, due to overfishing, global fishery resources have been on the verge of recession, which has also induced many worries about the sustainable consumption of fishery products. To achieve the sustainable development of the fishery and meet the growing demand for fishery products, many countries have proposed several regulations on the fishing [2] and started encouraging aquaculture [3]. In practice, although mariculture has a certain negative environmental impact, it has played an important role in ensuring food safety and promoting rural economic development in most countries [4].

However, mariculture is a risky production activity. According to questionnaire surveys in Norway [5], Denmark [6], Vietnam [7, 8], Bangladesh [9], France [10] and Thailand [11], the risk factors of mariculture yield are very complex, including weather, pollution disease and so on. These risk factors can not only endanger the stability of mariculture yield but can also cause serious social problems. Once the yield risk occurs, mariculture farmers may trap poverty because of the huge losses. In response to yield risk, many countries have launched mariculture insurance projects.

However, the failure of mariculture insurance has been widespread in many countries. Some scholars have evaluated the performance of aquaculture insurance programs in Asia, Europe, North America, South America, Africa, and Oceania [12]. They have found that aquaculture insurance in these areas, except for Europe, was not effective. Moreover, insurance programs focusing on mariculture species are worse. As of 2017, there were no pilot projects for mariculture species in India [13]. In Vietnam, Bangladesh, Norway, and other countries, mariculture insurance is also limited to a few species [14, 15].

There are many explanations for the failure of mariculture insurance, such as systemic risk, moral hazard, adverse selection, and high transaction costs [14]. However, the most direct reason still lies in unsound premium rates and pricing approaches [16]. The pricing principle of mariculture insurance is similar to that of crop insurance. In crop insurance projects, parametric distribution fitting and nonparametric distribution fitting are two common pricing approaches [17, 18]. According to the principle of reciprocity, the actuarially fair premium rate should be equal to the expected loss rate. These two methods use large sample data to fit the distribution of crop yield and solve the expected loss rate.

The parametric distribution fitting first assumes prior distribution of yield and then uses the maximum likelihood estimation to solve the parameters of the distribution. In the early stage, scholars believed that crop yield follows normal distribution [19]. However, empirical evidences show that the skewness and kurtosis of crop yield are inconsistent with the assumption of normal distribution. Scholars found that Beta distribution [20–24], Gamma distribution [25], Lognormal distribution [26] and Weibull distribution [27] are more suitable for fitting crop yield because of their wider ranges of kurtosis and skewness. Sherrick et al. (2004) argued that distribution of yield is very complex and goodness-of-fit statistics should be used to select the optimal distribution [27]. The nonparametric method does not assume prior distribution and often uses the kernel density estimation to fit [17, 28]. The two methods have no strict advantages and disadvantages. When the prior distribution is correct, the result of parametric method is more accurate. The nonparametric method is more flexible however, it cannot describe extreme events [29]. Many scholars have used these two methods to determine the premium rate in the crop insurance [30–33].

However, effective distribution fitting is based on long-term yield data, but a lack of reliable actuarial data is a major challenge in mariculture insurance premium pricing. In many developing countries, statistics on mariculture yield, sales, profits, and losses are extremely incomplete [15, 34]. Moreover, the cost of data collection for mariculturist can be high [14]. In the case of small samples, it is almost impossible to use distribution fitting for probability analysis. Hence, there was an absence of effective rules for the determination of premium rate in many countries, including China, Vietnam, Norway, India and Bangladesh [16, 34]. In practice, insurance companies prefer to use a simplified method to calculate the premium rate, that is, the empirical rate (ER). The China Fisheries Mutual Insurance association (CFMI), a leading mariculture insurance insurer, has explicitly used empirical rates as its main pricing method. The empirical rate is a simple average of the yield loss rate. However, an actuarially fair premium rate should be equal to the expected loss rate. Therefore, empirical rates can be biased, resulting in a high loss ratio for mariculture insurance projects. How to determine the premium rate in the case of small samples is a very important issue for mariculture insurance.

The purpose of this paper is to provide an improved method for premium computation of mariculture insurance using an IDM. The IDM was proposed by Huang [35] and was originally applied to the prediction of seismic probability. It was developed in function learning from a small sample of data [36]. With the improvement of many scholars, the IDM is now widely used in the field of risk assessment. For example, Olya and Alipour (2015) employed the IDM to estimate the risk of potential changes in the tourism climate [37]. Some scholars used the IDM to assess the risk of Alzheimer’s disease [38]. The IDM has also been introduced in the assessment of crop yield risk [39, 40]. Furthermore, researchers have frequently applied this approach to risk analysis of natural hazards, such as algal bloom outbreaks [41], hurricane [42], flood [43, 44], drought [45, 46], and grassland biological disasters [47, 48].

In the absence of widely accepted methods, the contribution of this paper is to provide an improved method for mariculture insurance pricing. We considered China’s oyster yield insurance as an example to show the calculation process, while we compared the difference between the rate calculated by the IDM and the empirical rate. We found that, compared with the empirical rate, the IDM could greatly improve the accuracy of premium rate calculation, especially in cases of small samples.

## Background

Like crop insurance, mariculture insurance provides a guarantee for the yield of mariculturists. However, the failure of the mariculture insurance market is more serious than that of crop insurance. The main reasons for the failure of mariculture insurance are as follows.

First, mariculture faces more complex risk factors than crops [49]. Typhoons, tsunamis, red tides, temperatures, salinity, disease, pollution, and many other harmful factors can cause yield loss. Moreover, risk factors such as marine disasters are almost impossible to avoid. The production risk of mariculture is significantly higher than that of crops [50]. Second, systematic risks, such as bad weather, are prone to occur, and the law of large numbers no longer works. Third, because the production risk of mariculture is high, the corresponding premium rate is also very high. At the same time, because of the high economic value of seafood, insurance liability is also higher than that of crops. Therefore, the premiums and compensation for mariculture insurance are higher. If an insurance company is not well managed, it could be responsible for enormous compensation. Smaller insurers could face bankruptcy. Fourth, the complexity of risk factors makes it difficult for insurance companies to distinguish exactly how losses are caused. Without regulating breeding logs, moral hazard is difficult to control [12]. Fifth, the subject matter of mariculture insurance is water, which is difficult to observe directly. When it comes to disasters, it is difficult for insurance companies to quantify the loss. Loss assessment can only be inferred by random sampling and has a high transaction cost. Sixth, mariculture insurance is still immature in many developing countries, and the ability of the insurer is not competent [51]. Seventh, the data on the production, sale, profit, and loss of mariculturists are incomplete. In the cases of small samples, the traditional pricing method is unsound [16]. Eighth, there is a lack of large reinsurance companies to cover risks [12].

Therefore, the promotion of mariculture insurance must overcome many difficulties, requiring the combined efforts of governments, insurance companies, economists, and aquatic scientists. This paper focuses on the key problem of how to calculate the premium rate in a small sample.

## Material and methods

### Data

This paper uses China’s oyster yield insurance as an example to demonstrate the calculation process. China is the world’s largest mariculture country, accounting for more than 60% of the total yield. However, China’s mariculture insurance is also facing serious market failure. In 1995, the People’s Insurance Company of China (PICC) started the pilot of the mariculture insurance project. However, this insurance pilot was forced to terminate in 1996 due to overly high compensation. According to statistics, the loss ratio of the entire project has reached approximately 200%. Under the joint initiative of the China Fisheries Mutual Insurance (CFMI) association, commercial insurance companies and farming cooperatives, the mariculture insurance project was relaunched in 2012.

Oysters are the mariculture species with the largest cultivation area in China. In 2017, the area of oyster farming reached 4,879,422 hectares. Liaoning (LN), Jiangsu (JS), Zhejiang (ZJ), Fujian (FJ), Shandong (SD), Guangdong (GD), and Guangxi (GX) are the main areas for oyster farming. To calculate the premium rate, we must obtain two variables: oyster yield and cultivation area. The data are from the China Fisheries Yearbook and the China Fisheries Statistical Yearbook. The time span of the data is 2003–2017, which is also the longest span of official data on oyster yield and cultivation area in China.

**Fig 1:**
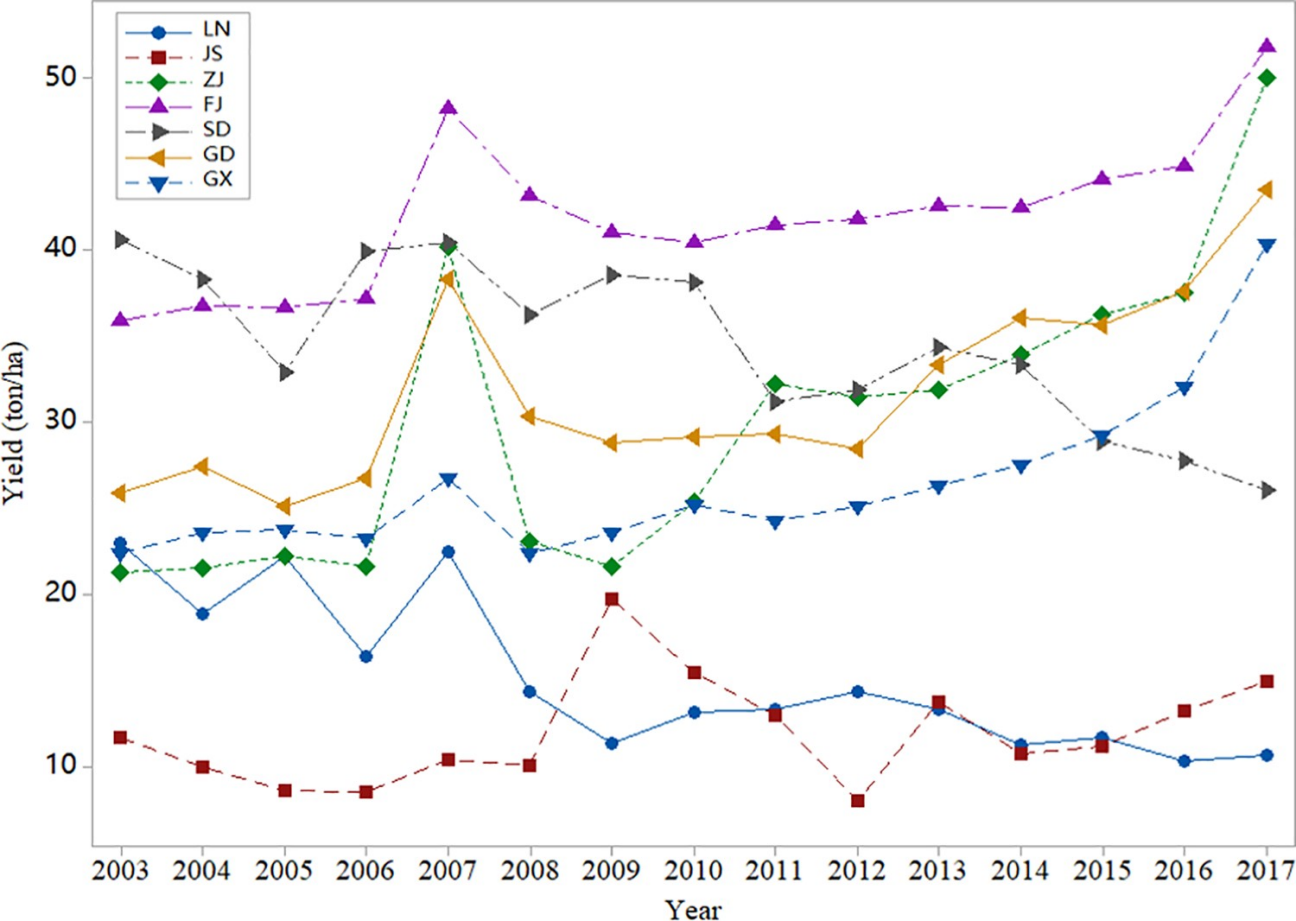
Oyster yield in seven provinces from 2003 to 2017 (ton/ha).

As shown in Fig 1, Fujian has the highest yield level, while Jiangsu has the lowest yield level. Moreover, the yield trend in these areas is not consistent. The yield in Fujian and Guangdong is on the rise, while the yield in Shandong and Liaoning provinces shows a certain downward trend. In addition, China’s oyster yield fluctuated greatly, especially in the provinces of Jiangsu and Liaoning, showing a sharp decline in 2007.

### Traditional premium rate pricing

Consider a common yield insurance contract. Assuming that the actual yield in year *t* is *y*_*t*_, the expected yield (trend yield) is *g*_*t*_ and the coverage is *λ*, then the loss rate in year *t* is:

$$x_{t}=\max(\frac{\lambda g_{t}-y_{t}}{\lambda g_{t}},0)\;t=1,2\ldots ,m$$ (1)

For simplicity, let us consider the case of *λ* = 1. If the probability density function of *x*_*t*_ is *f*(*x*), then the premium rate R is:

$$R=\int_{0}^{1}xf(x)dx$$ (2)

Therefore, the key to premium rate calculation is to scientifically solve the parameters of *f*(*x*). The most common methods in the literature are parametric distribution fitting and nonparametric distribution fitting [17, 52].

The parametric distribution fitting first assumes the prior distribution of *f*(*x*) and then solves the parameter through maximum likelihood estimation. However, two reasons prevent us from considering parametric distribution fitting. First, there is no literature examining the prior distribution of mariculture yield loss rates. Second, in the case of small samples, the maximum likelihood estimation might not be able to solve the distribution parameters. Relatively speaking, the result of nonparametric distribution fitting could be more reliable because it is relatively flexible.

Nonparametric distribution fitting is used to use the kernel density estimation (KDE) to fit the distribution. Kernel density estimation includes two types: fixed (global) bandwidth and varying (local) bandwidth. The kernel density estimation with fixed bandwidth is:

$$f(x)=\frac{1}{nh}\sum_{i=1}^{n}k[\frac{x-x_{i}}{h}]$$ (3)

where *x*_*i*_ is the sample, *n* is the sample size, and *k*(⋅) is a specific kernel function. In this paper, we choose the commonly used Gauss kernel function. *h* is the bandwidth. According to Silverman’s rule of thumb [53], the optimal bandwidth of the Gaussian kernel function is:

$$h=0.9\times \min\{\mathrm{standard}\;\mathrm{deviation},\frac{\mathrm{interquartile}\;\mathrm{range}}{1.34}\}\times n^{-\frac{1}{5}}$$ (4)

However, Ker and Goodwin [28] believed that kernel density estimation with fixed bandwidth could yield too many spurious details in the calculation of the premium rate. Because the data on the yield loss rate are not uniform, the fixed bandwidth approach can result in undersmoothing in areas with sparse observations. They proposed kernel density estimation with varying (local) bandwidth to improve the accuracy of premium rate calculation. This approach is also called adaptive kernel density estimation (AKDE). The formula is:

$$f(x)=\frac{1}{nh\lambda_{i}}\sum_{i=1}^{n}k[\frac{x-x_{i}}{h\lambda_{i}}]$$ (5)

where *λ*_*i*_ is the local bandwidth factor. *h* controls the overall degree of smoothing, while *λ*_*i*_ stretches or shrinks the sample point bandwidths to adapt to the density of the data [54]. *λ*_*i*_ can be calculated by the following equation:

$$\lambda_{i}={\left(\frac{f(x_{i}{)}^{\prime }}{G}\right)}^{-\alpha }$$ (6)

where *f*(*x*_*i*_)′ is a standard fixed bandwidth kernel density estimate obtained with *h* as the bandwidth. *G* is the geometric mean over all *i* of the pilot density estimate *f*(*x*_*i*_)′. *α*∈[0, 1] is the sensitivity parameter. When *α* = 0, it reduces to the standard kernel density estimation. We set *α* = 0.5, as suggested by Van Kerm [54].

For convenience, the insurance premium rates calculated by standard (fixed bandwidth) kernel density estimation and adaptive kernel density estimation are denoted as *R*_*KDE*_ and *R*_*AKDE*_, respectively.

In general, sufficient data is required to calculate premium rates using these two methods. In the field of crop insurance, most of literature use the about 15 years of loss observations to calculate the premium rate [17, 32, 52]. However, in practice, due to the unavailability of sample data, insurance companies prefer to use the empirical rate. The empirical rate method simply denotes average loss rates as the premium rate *R*_*ER*_:

$$R_{ER}=\frac{x_{1}+x_{2}+\ldots +x_{m}}{m}$$ (7)

Obviously, this approach is inaccurate because the actuarially fair premium rate should be equal to the expected loss rate, not the mean loss rate. However, in the absence of a widely accepted method, the China Fisheries Mutual Insurance Association mostly adopts this approach.

### Premium rate pricing based on the IDM

According to information diffusion theory, when the sample size is small, the information provided by the sample is incomplete. The information of a sample should not be regarded as the exact information but should be regarded as representative of the sample and a fuzzy observation sample. In this case, it might be inappropriate to use statistical methods to infer probabilities [55]. The purpose of information diffusion is to excavate as much useful information as possible from the incomplete sample and enhance the accuracy of system identification [47].

Let *X* = {*x*_1_,*x*_2_,…,*x*_*m*_} be a given sample to estimate the relationship *R* of the universe *U* = {*u*_1_,*u*_2_,…*u*_*j*_…,*u*_*n*_}. Huang proved that there must be reasonable information diffusion functions to improve the nondiffusion estimator if and only if *X* is incomplete [35], which means that, when *X* is incomplete, there must be some way to be able to obtain fuzzy information about *X* to more accurately estimate the function approximation of a relation *R*.

Normal diffusion is the most common form. The sample *x*_*i*_ diffused its information to all of the points of the set *U* based on Eq (8).

$$f_{i}(u_{j})=\frac{1}{h\sqrt{2\pi }}\exp[-\frac{{\left(x_{i}-u_{j}\right)}^{2}}{2h^{2}}]$$ (8)

where *h* is the diffusion coefficient. According to the findings of the previous literature [37, 42, 56], *h* can be calculated through Eq (9):

$$h=\{\begin{array}{l} 1.6987(b-a)/(m-1)\;1<m\leq 5\\ 1.4456(b-a)/(m-1)\;6\leq m\leq 7\\ 1.4230(b-a)/(m-1)\;8\leq m\leq 9\\ 1.4208(b-a)/(m-1)\;10\leq m \end{array}$$ (9)

where *b* and *a* are the maximum and minimum values of sample *x*, respectively. In practice, *U* represents the loss rate of mariculture yield, obviously *U*∈[0,1]. *U* can be set as *U* = {0,0.02,0.04,…,1}. To ensure that each diffusion sample has the same status for the evaluation results, Eq (8) must be normalized. The normalized diffusion vector can be defined as:

$$u_{x_{i}}(u_{j})=\frac{f_{i}(u_{j})}{\sum_{j=1}^{n}f_{i}(u_{j})}$$ (10)

Through the above steps, we can change the sample *X* = {*x*_1_,*x*_2_,…,*x*_*m*_} into a fuzzy subset with $u_{x_{i}}(u_{j})$ as the membership function. The probability of loss rate *U* = {0,0.02,0.04,…,1} can be calculated by frequency. Based on Eq (10), we can obtain

$$q(u_{j})=\sum_{i=1}^{m}u_{x}{}_{{}_{i}}(u_{j})$$ (11)

Eq (11) means that, when *x*_*i*_ is regarded as representative of samples, after information diffusion, the loss rate *u*_*j*_ is *q*(*u*_*j*_).

By totaling the number of samples at each *u*_*j*_, we can obtain:

$$Q=\sum_{j=1}^{n}q(u_{j})$$ (12)

*Q* is theoretically equal to *m*. Thus, the probability of loss rate *u*_*j*_ is:

$$p(u_{j})=\frac{q(u_{j})}{Q}=\frac{q(u_{j})}{m}$$ (13)

Hence, under the framework of information diffusion theory, the premium rate *R*_*IDM*_ should be:

$$R_{IDM}=\sum p(u_{j})\times u_{j}$$ (14)

## Results

Regardless of the method used, the yield loss rate must be calculated first. In crop insurance projects, it is generally assumed that yield has a positive relationship with the time trend [18, 57]. However, Fig 1 shows that mariculture yield fluctuates greatly and is not necessarily related to the time trend. In this paper, the HP filter is used to decompose the expected yield. This method was proposed by Hodrick and Prescott [58] and was originally used to study the economic fluctuations of postwar America. It has been widely used in the field of yield fluctuation. The HP filter assumes that the yield sequence *y*_*t*_ is composed of trend term *g*_*t*_ and fluctuation term *c*_*t*_. The trend term is determined by minimizing the loss function:

$$\begin{array}{c} \min\\ {\left\{g_{t}\right\}}_{t=-1}^{T} \end{array}\{\sum_{t=1}^{T}{\left(y_{t}-g_{t}\right)}^{2}+\theta \sum_{t=1}^{T}{\left[(g_{t}-g_{t-1})-(g_{t-1}-g_{t-2})\right]}^{2}\}$$ (15)

where *θ* is the conversion factor, and the second term in Eq (15) is mainly used to adjust the smoothness of the trend term. Eq (16) can be obtained from Eq (15) as follows:

$$c_{t}=\theta Fg_{t}$$ (16)

where F is a constant coefficient matrix of a fixed T×T. Combining *y*_*t*_ = *g*_*t*_+*c*_*t*_, we can solve the trend term:

$$g_{t}={\left(\theta F+I\right)}^{-1}y_{t}$$ (17)

**Fig 2:**
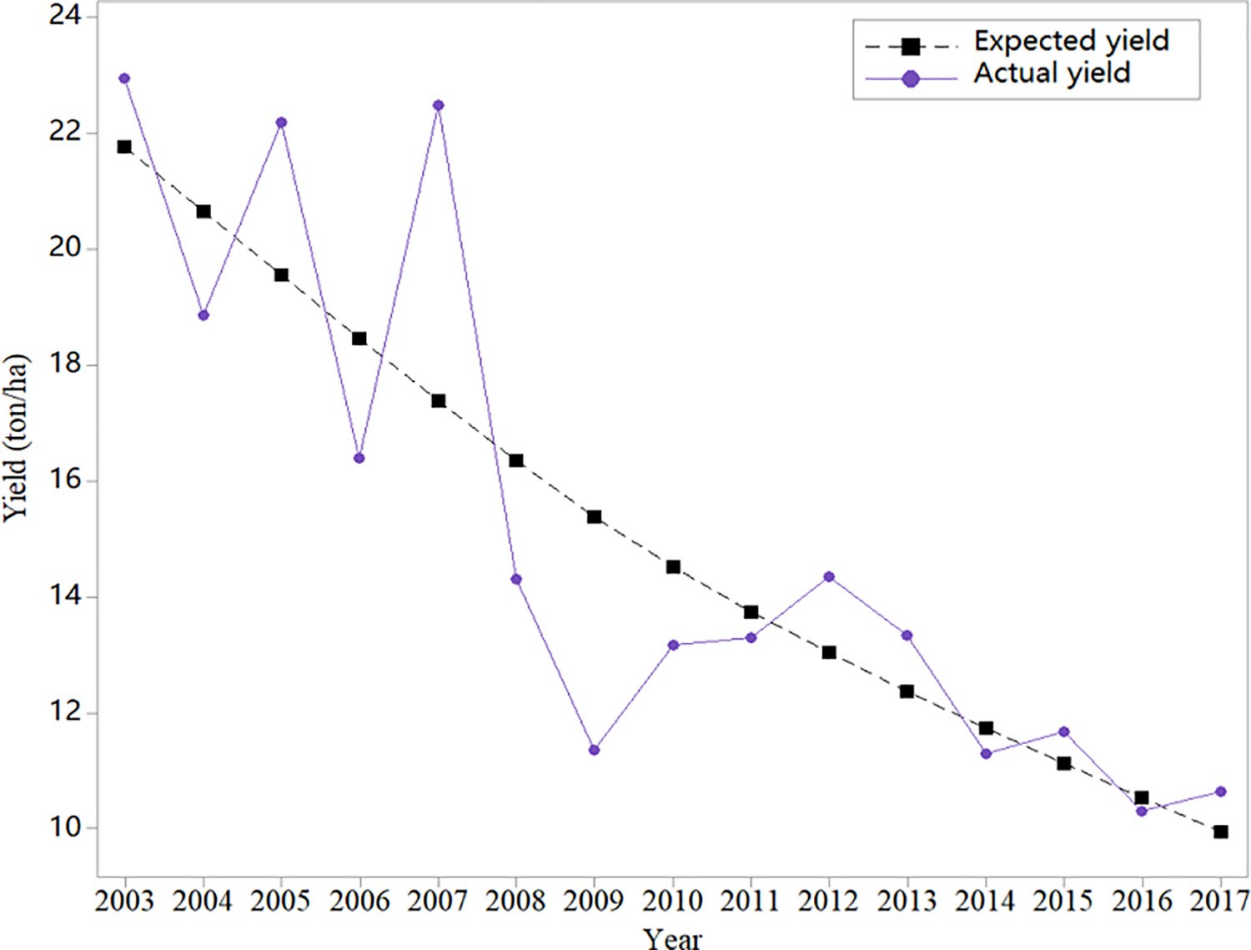
Actual and expected oyster yield in Liaoning province.

According to experience, when annual data are used, the best fitting effect is *λ* = 100. Fig 2 shows the expected yield of oysters in Hebei Province using the HP filter. This method perfectly captures the downward trend of oyster yield in Hebei Province.

According to the HP filter and Eq (1), the yield loss rate of 7 provinces in 2003–2017 is shown in Table 1. Overall, the loss rates of oyster yield in these provinces are not high, and most of them are less than 10%. In addition, the loss rates of high-yield areas such as Fujian and Shandong were smaller, while those of low-yield areas such as Liaoning and Jiangsu were higher. This outcome might occur because, in high-yield areas, farming technology is more advanced, and the ability to resist risks is stronger.

**Table 1:** Loss rate of oyster yield in 2003–2017.

Year	LN	JS	ZJ	FJ	SD	GD	GX
2003	0.000	0.000	0.000	0.021	0.000	0.008	0.000
2004	0.087	0.030	0.020	0.022	0.022	0.000	0.000
2005	0.000	0.194	0.037	0.046	0.148	0.089	0.000
2006	0.112	0.226	0.106	0.053	0.000	0.052	0.000
2007	0.000	0.090	0.000	0.000	0.000	0.000	0.000
2008	0.125	0.148	0.124	0.000	0.018	0.000	0.066
2009	0.261	0.000	0.216	0.006	0.000	0.045	0.036
2010	0.092	0.000	0.120	0.034	0.000	0.057	0.000
2011	0.033	0.000	0.000	0.023	0.092	0.075	0.062
2012	0.000	0.363	0.013	0.027	0.045	0.129	0.065
2013	0.000	0.000	0.055	0.027	0.000	0.016	0.059
2014	0.039	0.159	0.051	0.045	0.000	0.000	0.063
2015	0.000	0.133	0.042	0.029	0.034	0.024	0.052
2016	0.022	0.000	0.064	0.031	0.029	0.010	0.012
2017	0.000	0.000	0.000	0.000	0.048	0.000	0.000

**Fig 3:**
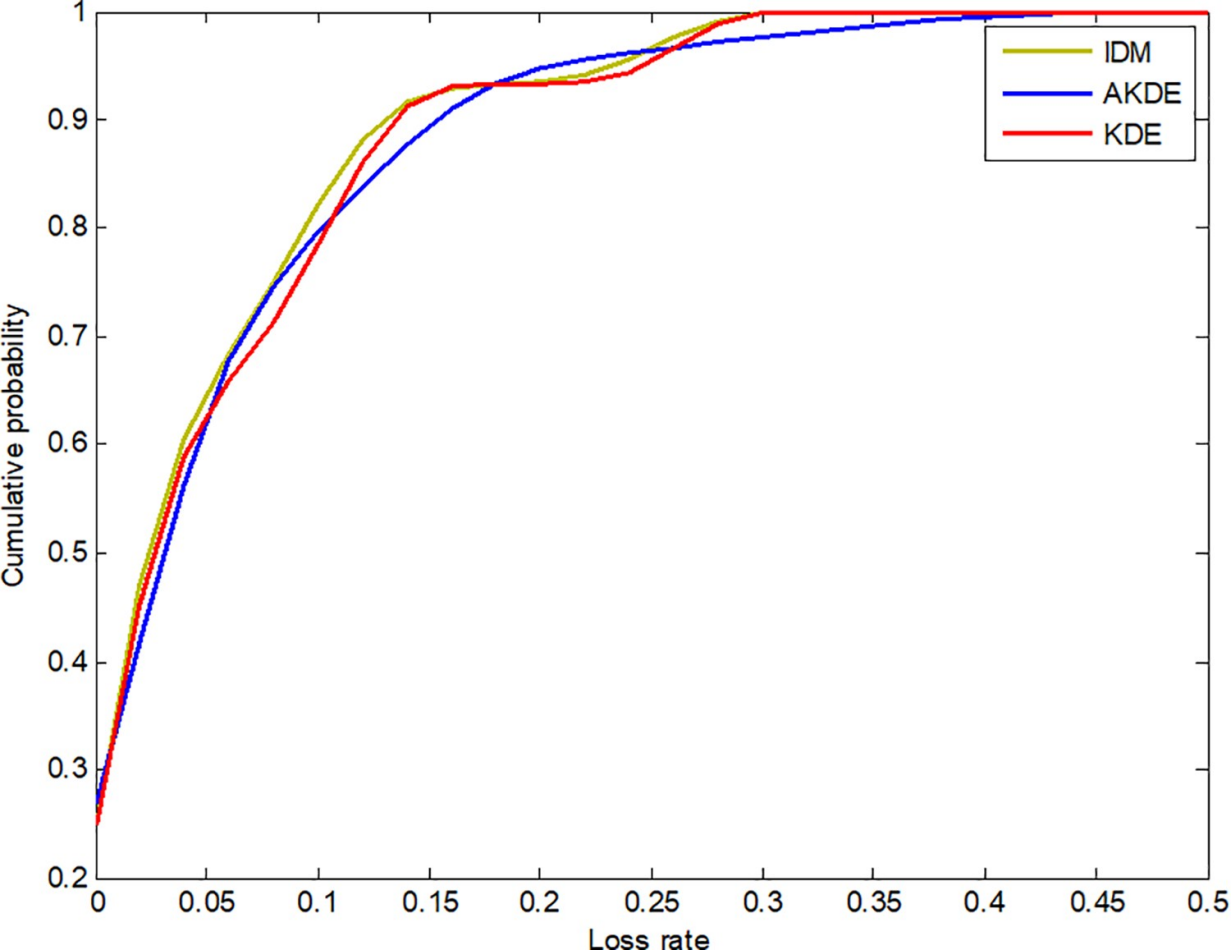
Cumulative probability of loss rate in Liaoning province.

Next, we compare the differences in insurance premium rates calculated by the four methods. First, consider the case in which the loss rate data are available from 2003 to 2017. Although the 15-year data do not constitute a large sample, they reflect sufficient information. In this case, it can be considered that the premium rate calculated by kernel density estimation is relatively reasonable. Of course, in theory, the results of AKDE are more accurate than those of KDE. Considering Liaoning as an example, Fig 3 shows the cumulative probability of the loss rate fitted by KDE and AKDE. The curve fitted by AKDE is smoother than that fitted by KDE. Hence, we can judge the performance of IDM by comparing the difference between *R*_*IDM*_ and *R*_*AKDE*_. The results are shown in Table 2.

**Table 2:** Premium rates for oyster yield insurance in seven provinces.

Province	ER	IDM	KDE	AKDE
LN	5.14%	5.93%	5.88%	5.86%
JS	8.95%	10.12%	10.13%	10.08%
ZJ	5.65%	6.04%	6.12%	6.14%
FJ	2.42%	2.30%	2.51%	2.64%
SD	2.91%	3.22%	3.23%	3.29%
GD	3.36%	3.56%	3.73%	3.77%
GX	2.76%	2.76%	3.07%	3.16%

First, we found that the results of IDM were very close to those of AKDE. The difference between *R*_*IDM*_ and *R*_*AKDE*_ is also very small. In addition, Fig 3 shows that, although the IDM fitted curve is not as smooth as AKDE, it is better than KDE. This outcome means that IDM is comparable to traditional nonparametric distribution fitting. However, there is a large gap between *R*_*ER*_ and *R*_*AKDE*_. In comparison, the empirical rate *R*_*ER*_ is lower than the premium rate *R*_*IDM*_ and *R*_*AKDE*_. In theory, the rate calculated by AKDE can be regarded as an actuarially fair rate, indicating that the empirical rate is smaller than the actuarially fair premium rate. This underestimation also explains the high ratios of mariculture insurance projects in China, also suggesting that IDM performs better than ER.

Of course, in the above samples, ER is not as good as IDM, but the gap is still acceptable. However, in cases of small samples, the gap between the two will be obvious. Let us illustrate this point by calculating the rate in the case of a small sample.

**Fig 4:**
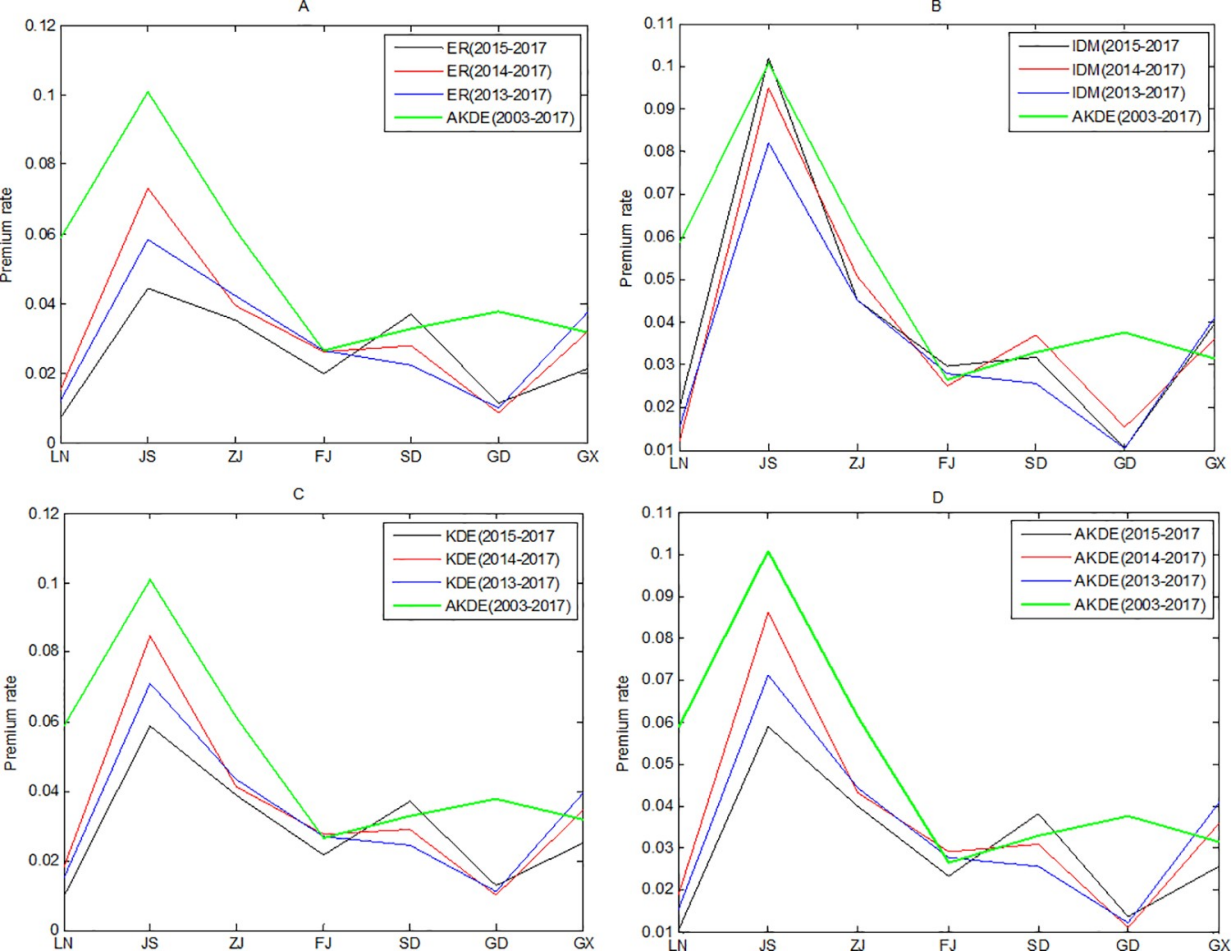
Premium rate calculated by four methods in cases of small samples.

We regard the premium rate calculated by AKDE in Table 2 as the actuarially fair premium rate. Suppose that we only know the loss rate of mariculture yield in recent years. We then calculate the corresponding rates through different methods. To better reflect the advantages of IDM in small samples, we also calculate the results of KDE and AKDE in small samples. Because there is no standard production record, insurers may only obtain the recent 3- to 5-year loss rate data of mariculture households. Therefore, the premium rates were calculated for sample sizes of 3, 4, and 5 years, corresponding to the data available from 2015 to 2017, from 2014 to 2017 and from 2013 to 2017, respectively. The results are shown in Fig 4.

Intuitively, it can be found that the results of IDM were more stable than those of the other three methods with small samples. Under different sample sizes, *R*_*IDM*_ does not fluctuate much. Moreover, it can be found that, in cases of small samples, the rate measured by IDM is generally higher and is closer to actuarially fair rates. This outcome is most obvious in Jiangsu and Zhejiang provinces.

Table 3 provides more specific results. Except for Guangdong Province, the rate calculated by IDM is better than the other three methods in accuracy and stability. In addition, kernel density estimation does not significantly improve the empirical rate for small samples. This outcome is also in line with theoretical expectation. In cases of small samples, using statistical theory to infer probability might be inefficient. If an insurer adopts the empirical rate, the indemnity payment will be much larger than the premium, resulting in a higher loss ratio.

**Table 3:** Mean and standard deviation of the rate calculated by the four methods.

	ER		IDM		KDE		AKDE		PM
	Mean	SD	Mean	SD	Mean	SD	Mean	SD	
LN	1.16%	0.40%	1.60%	0.41%	1.42%	0.42%	1.49%	0.42%	IDM
JS	5.86%	1.44%	9.29%	1.00%	7.14%	1.30%	7.21%	1.35%	IDM
ZJ	3.90%	0.36%	4.70%	0.32%	4.10%	0.24%	4.25%	0.21%	IDM
FJ	2.42%	0.37%	2.76%	0.24%	2.54%	0.32%	2.67%	0.31%	IDM
SD	2.90%	0.75%	3.15%	0.57%	3.02%	0.63%	3.16%	0.63%	IDM
GD	0.99%	0.14%	1.21%	0.29%	1.13%	0.13%	1.23%	0.14%	AKDE
GX	3.01%	0.81%	3.89%	0.25%	3.30%	0.73%	3.42%	0.77%	IDM

Note: SD: standard deviation; PM: preferred model.

Of course, it must be explained why IDM is not ideal for calculating insurance rates for Guangdong Province. Indeed, the premium rates in Liaoning Province also differ greatly from actuarially fair rates, mainly because, in the sample period, the oyster yield in Liaoning and Guangdong seldom decreased. In this case, it is difficult to extract useful information through sample diffusion. However, this fact is not an inherent defect of IDM. Probability inference is almost impossible without valid information.

In summary, the example shows that, compared with the empirical rate, the IDM can improve the accuracy of mariculture insurance premium rates. This finding is essential to improving the performance of insurance programs and promoting the sustainable development of mariculture insurance.

## Discussion and conclusion

### Key findings

Mariculture insurance is an important tool for managing mariculture yield risk, but the failure of the insurance market is widespread worldwide. Unsound premium rates and pricing methods are important reasons hindering the development of mariculture insurance. In practice, due to the lack of long-term historical yield data, insurance companies often simply use the empirical rate as an actuarially fair premium rate. In cases of small samples, this approach is likely to result in an underestimation of the premium rate and high loss ratios. The contribution of this paper is to provide an improved method for premium computation of mariculture insurance using an IDM. This analysis is of interest since there are currently no widely accepted methods for modeling and computing mariculture insurance premium rate. Two key findings in this paper are summarized below.

On the one hand, the results demonstrated the practicality of the IDM in determining mariculture insurance premium rates. Even in the case of sufficient samples, the performance of IDM is comparable to traditional distribution fitting. Other studies have also shown that the IDM can extract enough useful information for probability analysis [37–40]. Combining fuzzy mathematics and probability theory, IDM has a very solid theoretical foundation [36, 38]. The simple and objective calculation process of the IDM will also increase the feasibility in practice. These advantages make the IDM very useful in the natural, social, medical and other fields of risk analysis [37, 47].

On the other hand, the results also show that, in cases of small samples, IDM is superior to the empirical rate and kernel density estimation in accuracy and stability. The traditional kernel density estimation is inefficient and does not significantly improve the empirical rates. The empirical rate method is only a simplified calculation method, which is not actuarial fair in theory. Meanwhile, the finding also confirmed the inference that without sufficient information, kernel density estimation is inefficient [55, 59]. Previous studies have also demonstrated the superiority of IDM for risk analysis in an uncertain, random, and complex system. For example, Lu et al. (2014) found that compared with the kernel density estimation, the results of information diffusion technology have good consistency and continuity in the analysis of grassland biological disaster risk [47]. Li et al. (2014) also demonstrated that IDM is more stable and effective than the traditional distribution fitting method in flood risk assessment [44].

The reason why the IDM performs better in the case of small samples is that it can make up for the information blanks caused by incomplete data through effective information diffusion. It is especially suitable for mariculture insurance with imperfect data records. Therefore, this paper suggests that insurers gradually abandon the empirical rate method and try to use the IDM to improve the performance of mariculture insurance projects.

Of course, the risk management of mariculture should be diversified. In addition to insurance, disaster prevention also needs to be strengthened. In addition to short-term risk factors such as weather, disease and pollution, the long-term risk of climate can not be ignored. Climate change will affect the temperature and salinity of seawater, which may cause the loss of mariculture [60]. Therefore, effective mariculture management should be established to improve biological and socioeconomic resilience to climate change. Specific measures include diversifying the income of mariculture farmers, improving farming techniques and strengthening environmental monitoring [61]. In addition, weather derivatives such as futures and options can also be developed to manage the climate risks of mariculture.

### Future research

First, improve the IDM. Although the IDM has many advantages, it also has some shortcomings: (1) there is an absence of the principle of information diffusion function selection. In empirical analysis, normal information diffusion is generally selected; (2) The calculation of information diffusion coefficient is complicated. The calculation process requires extensive computer simulation. In future research, scholars can compare the performance of different information diffusion functions in the calculation of mariculture insurance premium rates, so as to select the most appropriate information diffusion function for mariculture insurance.

Second, compare IDM with more models. This paper only compares the difference between the IDM and the most commonly used methods in yield insurance. Methods of probability analysis also include bootstrap, Bayes, Monte Carlo simulations and so on. Whether the IDM is superior to these models remains to be analyzed.

Third, improve methods for calculating the expected yield. In determining the premium rate of mariculture insurance, not only the choice of probability analysis model is very important, but also the calculation of expected yield. This paper uses HP filter method to get the expected yield. Because of the complex risk factors involved in mariculture, the sequence of mariculture yield is extremely variable. Future research also needs to discuss appropriate methods for calculating the expected yield of mariculture.

## Supporting information

S1 Table Output of oysters from 2003 to 2017. (DOCX)

S2 Table Area of oysters from 2003 to 2017. (DOCX)
