# Supplementary material for: Improving mariculture insurance premium rate calculation using an information diffusion model
Source: PLoS One. 2021 Dec 23;16(12):e0261323. doi: 10.1371/journal.pone.0261323 (PMC8700043; doi:10.1371/journal.pone.0261323)
Supplement: S1 Table — (DOCX) [file pone.0261323.s001.docx]

**S1 Table. Output of oysters from 2003 to 2017(ton)**

| Year | LN | JS | ZJ | FJ | SD | GD | GX | HN |
| --- | --- | --- | --- | --- | --- | --- | --- | --- |
| 2003 | 203327 | 6885 | 124670 | 1622774 | 582146 | 656136 | 451263 | 8809 |
| 2004 | 184424 | 6500 | 122495 | 1674245 | 570155 | 711092 | 466569 | 8750 |
| 2005 | 209224 | 6256 | 118401 | 1706364 | 568961 | 734094 | 466710 | 3173 |
| 2006 | 179045 | 6422 | 117636 | 1685948 | 591376 | 816085 | 480494 | 3761 |
| 2007 | 141358 | 6295 | 113383 | 1441831 | 535826 | 870959 | 385949 | 383 |
| 2008 | 143545 | 6182 | 111229 | 1419083 | 498419 | 825102 | 345957 | 1398 |
| 2009 | 130715 | 10988 | 105235 | 1449537 | 581452 | 864274 | 360255 | 1326 |
| 2010 | 162345 | 11953 | 112906 | 1456106 | 566965 | 931945 | 399210 | 1349 |
| 2011 | 133431 | 29711 | 150392 | 1475485 | 575647 | 973961 | 416230 | 1423 |
| 2012 | 146636 | 39438 | 148687 | 1476422 | 669534 | 1027268 | 439295 | 1517 |
| 2013 | 201364 | 38811 | 154566 | 1521408 | 758177 | 1069148 | 473432 | 1738 |
| 2014 | 170533 | 39580 | 162402 | 1612385 | 803493 | 1079949 | 480322 | 3389 |
| 2015 | 184993 | 42129 | 169867 | 1659572 | 856837 | 1147916 | 508684 | 3372 |
| 2016 | 223501 | 51679 | 172374 | 1746591 | 872797 | 1221973 | 541956 | 3656 |
| 2017 | 226128 | 48185 | 195871 | 1788061 | 910685 | 1116515 | 589875 | 4102 |
