# Supplementary material for: Improving mariculture insurance premium rate calculation using an information diffusion model
Source: PLoS One. 2021 Dec 23;16(12):e0261323. doi: 10.1371/journal.pone.0261323 (PMC8700043; doi:10.1371/journal.pone.0261323)
Supplement: S2 Table — (DOCX) [file pone.0261323.s002.docx]

**S2 Table. Area of oysters from 2003 to 2017(ha)**

| Year | LN | JS | ZJ | FJ | SD | GD | GX | HN |
| --- | --- | --- | --- | --- | --- | --- | --- | --- |
| 2003 | 8862 | 590 | 5858 | 45182 | 14343 | 25309 | 20128 | 686 |
| 2004 | 9775 | 650 | 5683 | 45550 | 14894 | 25911 | 19802 | 713 |
| 2005 | 9429 | 729 | 5325 | 46527 | 17291 | 29242 | 19683 | 481 |
| 2006 | 10920 | 754 | 5439 | 45368 | 14820 | 30479 | 20647 | 438 |
| 2007 | 6286 | 607 | 2823 | 29925 | 13267 | 22732 | 14433 | 242 |
| 2008 | 10033 | 616 | 4818 | 32871 | 13756 | 27226 | 15431 | 299 |
| 2009 | 11505 | 556 | 4877 | 35324 | 15078 | 29981 | 15257 | 300 |
| 2010 | 12326 | 774 | 4454 | 36030 | 14879 | 32013 | 15813 | 313 |
| 2011 | 10037 | 2290 | 4664 | 35625 | 18448 | 33211 | 17140 | 318 |
| 2012 | 10218 | 4934 | 4722 | 35297 | 21009 | 36091 | 17495 | 344 |
| 2013 | 15082 | 2820 | 4852 | 35764 | 22056 | 32113 | 17967 | 360 |
| 2014 | 15108 | 3695 | 4793 | 37934 | 24095 | 29970 | 17474 | 284 |
| 2015 | 15845 | 3770 | 4687 | 37659 | 29653 | 32199 | 17395 | 280 |
| 2016 | 21672 | 3901 | 4592 | 38901 | 31395 | 32492 | 16906 | 295 |
| 2017 | 21241 | 3230 | 3920 | 34505 | 34953 | 25686 | 14622 | 305 |
